# Hard Palate Foreign Body in a Pediatric Patient: A Case Report

**DOI:** 10.7759/cureus.68474

**Published:** 2024-09-02

**Authors:** Meshal Atiyah

**Affiliations:** 1 Pediatric Emergency Medicine, Security Forces Hospital, Makkah, SAU

**Keywords:** pediatric, hard palate foreign body, foriegn body, pediatric foreign body removal, hard palate

## Abstract

Hard palate foreign bodies are rare and demonstrate a unique presentation involving minimal symptoms and irritation. Such foreign bodies can be difficult to remove due to variations in their shape and the anatomy of the palate. Here, we present the case of a four-year-old girl with a piece of metal embedded in her hard palate. The presentation was acute, and the foreign body was successfully removed. No complications were observed at the two-week follow-up. The patient was in good health and did not demonstrate deformities.

## Introduction

Pediatric foreign bodies are frequent causes of pediatric emergencies and are more common in curious younger children [1] due to inadequate parental supervision [2]. Attention deficit and hyperactivity disorder, autism spectrum disorder, and other mental conditions might also increase foreign body injury risk in pediatric patients [1]. Hard palate foreign bodies are rare [3], and their presentation is typically unusual; for example, the long-term presence of a metal foreign body in the absence of pain [3,4] or a foreign body mimicking a hard palate fistula [5]. Here, we present the case of a four-year-old girl with acute presentation of a hard foreign body in her hard palate.

## Case presentation

A four-year-old girl presented at the pediatric emergency department (ED) complaining of a foreign body in her hard palate three hours prior. She had an unremarkable medical history and mentioned that she placed the cap of a battery on her hard palate due to curiosity with no self-harm intentions. Her father mentioned previous attempts to remove the foreign body one hour before the presentation. However, these attempts failed, even with the full cooperation of the child. The patient denied any other complaints or pain.

**Figure 1:**
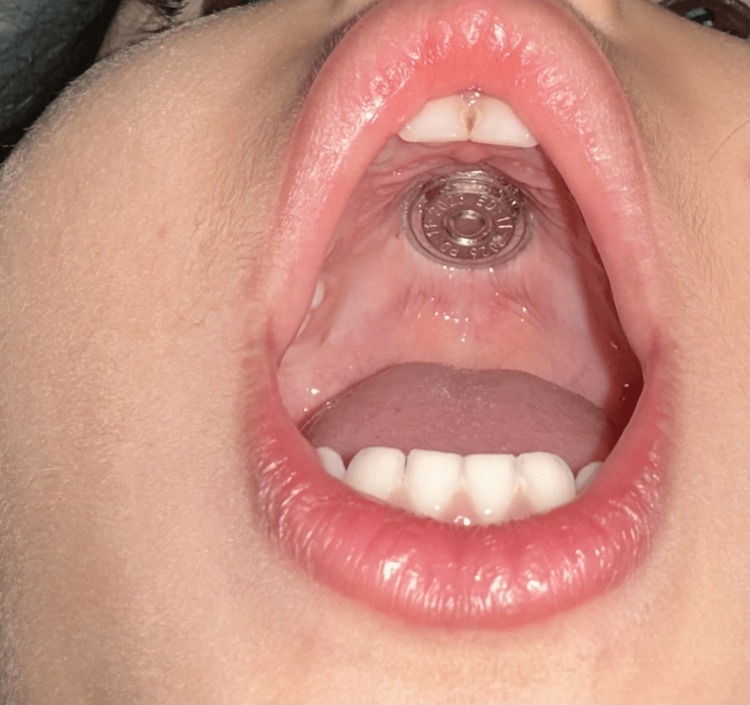
Embedded foreign body on the hard palate. Examination of the palatine rugae revealed a round, shiny metallic disc measuring 1.5 cm in diameter with clear edges.

**Figure 2:**
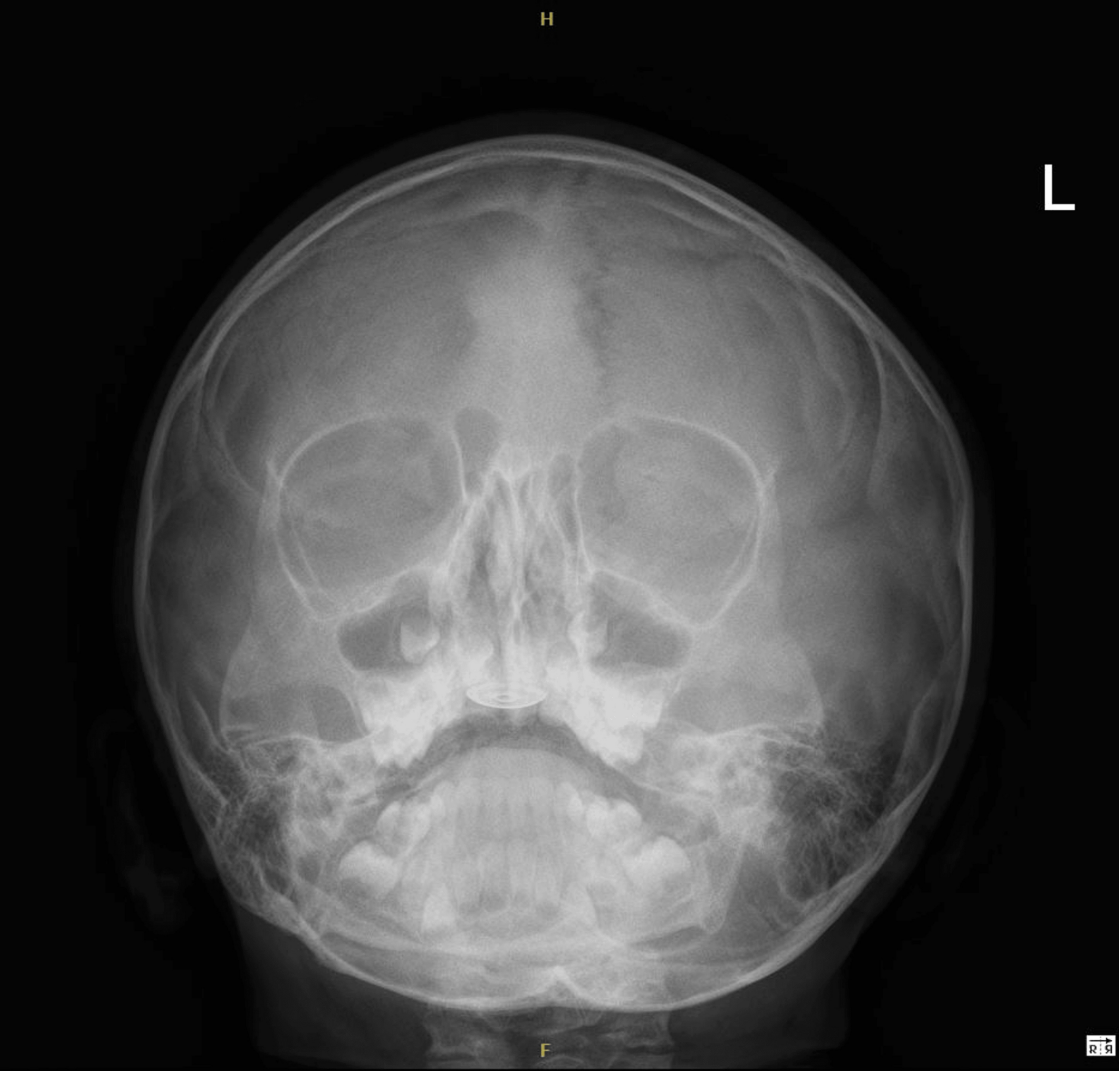
X-ray showing the flat foreign body. Skull X-ray, paranasal sinuses Waters view showing a round and flat radiopaque foreign body on the hard palate.

**Figure 3:**
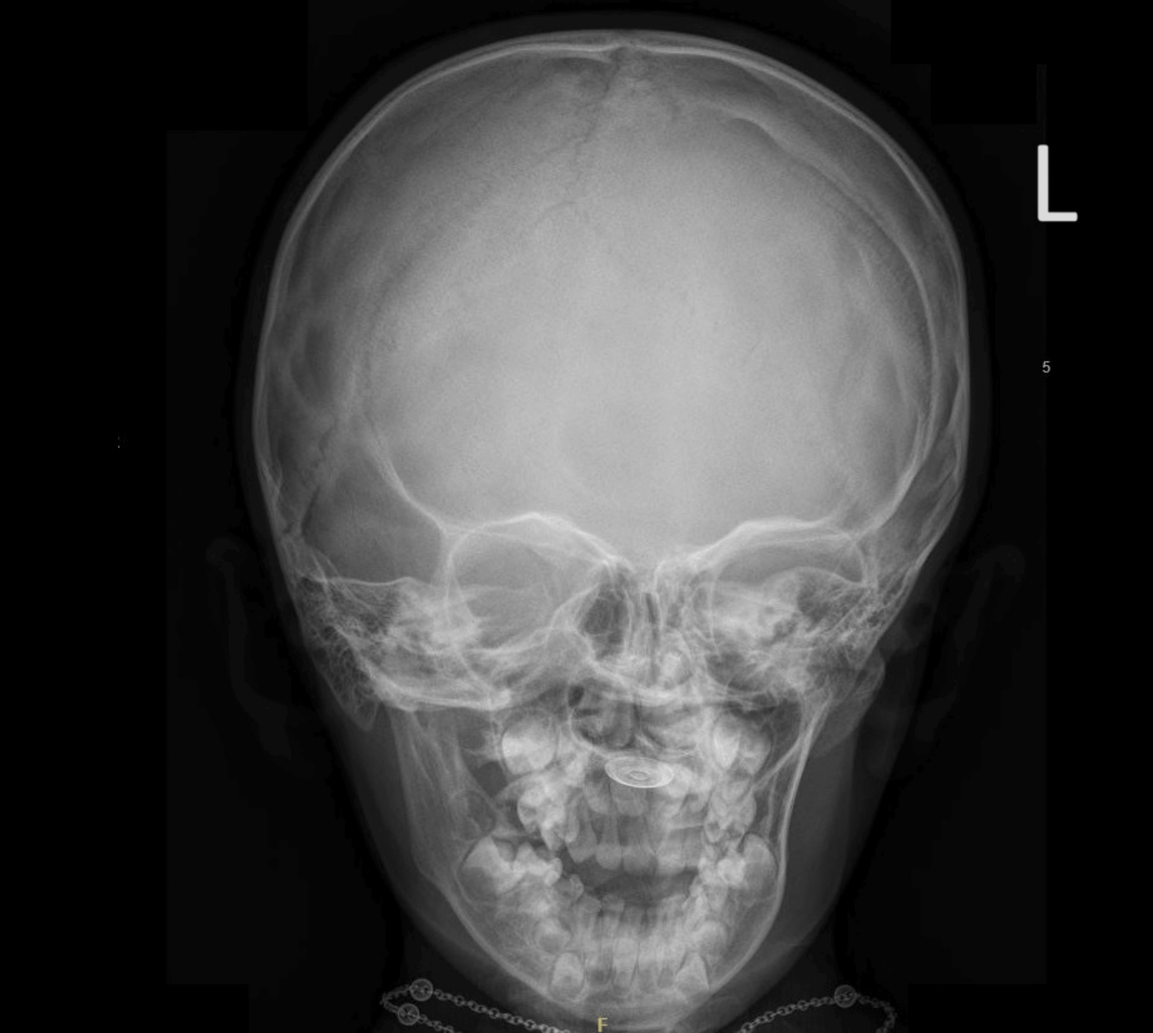
X-ray showing a round foreign body in the hard palate. Anteroposterior view skull X-ray showing a flat and round foreign body in the hard palate.

**Figure 4:**
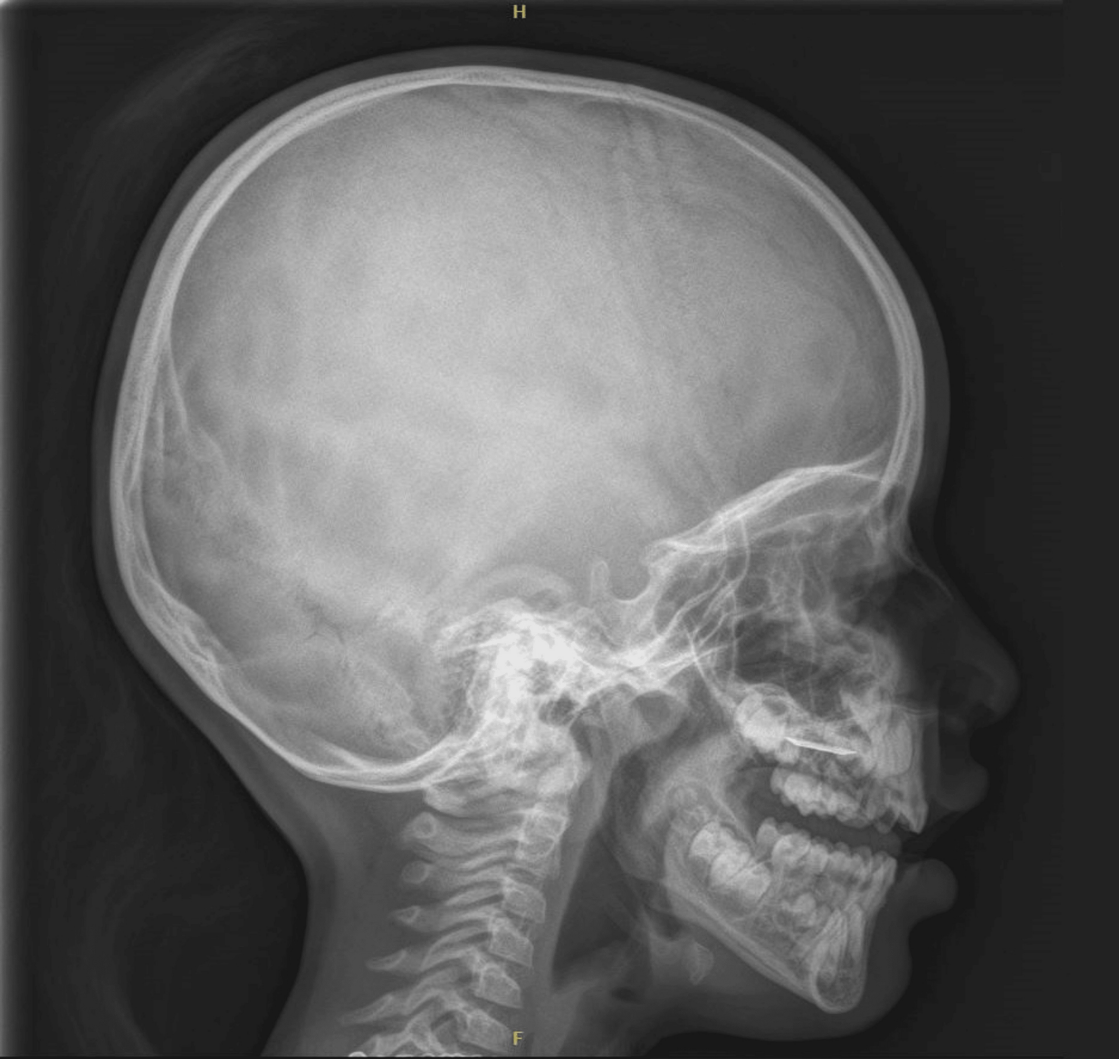
X-ray lateral view showing the foreign body. Lateral view skull X-ray showing a flat and round radiopaque foreign body located at the level of the hard palate.

On examination, a shiny metallic cap was noted embedded over the palatine rugae (Figure 1). The other parts of the hard and soft palates appeared normal. Examination of the palatine rugae revealed a round, shiny metallic disc measuring 1.5 cm in diameter with clear edges (Figure 1). The ear, nose, and throat examination results were normal. X-rays showed a flat, round, metallic foreign body in the hard palate (Figures 2-4). Multiple attempts to remove the foreign body at the ED, including approaches using toothed forceps and a tongue depressor, were unsuccessful. An otolaryngology consultation was conducted, and multiple attempts with a foreign body removal hook in the clinical setting failed. The child became uncooperative due to the discomfort she experienced during the removal attempts despite local anesthesia. Therefore, the decision was made to remove the foreign body under general anesthesia.

Preoperative preparation included laboratory investigation, anesthesia revision, removal of the metallic cap under general anesthesia, smooth intubation with no apparent airway abnormalities, and intubation with a transoral cuffed endotracheal tube (4, 5). Gentle removal of the foreign body using a periosteum elevator from the side of the metallic cap was sufficient, and no deep wounds or palate abnormalities were observed after removal. The area was clean after the foreign body removal; thus, no antibiotics were given. General instructions were provided to the parents regarding oral hygiene, and the child was discharged the same day after recovering from the anesthesia and tolerating her full feed. A follow-up examination was conducted in the clinic two weeks later, revealing no wounds, complaints, or pain.

## Discussion

Foreign bodies embedded in the hard palate are very rare and, when incidentally discovered, should be distinguished from congenital oral pathologies [5,6]. A few studies have described foreign bodies embedded in the hard palate [2,4], most in pediatric patients [6]. One previous case of an embedded metallic AA battery has been reported as this case was reviewed [4]. Reported hard palate foreign bodies include wood, nutshells, nails, coins, and reflective pieces of metal [2-5,7-9]. The foreign body may be present in the hard palate for a long period after impaction due to a lack of pain and irritation, unlike the presentation of other foreign bodies. The duration from impaction until presentation can be as high as 18 months [3,10] or limited to a few hours, as in our case and some others [11]. Previous attempts to remove hard palate-embedded foreign bodies by inexperienced personnel can worsen the situation and further embed the foreign body into the hard palate. As described in the literature, foreign body removal can be performed with local [7] or general [1] anesthesia in the clinic. General anesthesia was used for our patient because of the embedded nature of the foreign body, the previous attempts at removal, and the need to provide airway protection.

## Conclusions

Foreign bodies in the hard palate are rare; patients are usually pediatric. A lack of symptoms is common and may delay presentation. Meticulous physical examination is key in excluding foreign bodies and discovering the etiology of difficult cases with vague complaints. The removal technique varies according to the clinical situation, the nature of the foreign body, and the anesthesia used.
